# Complete Genome Sequences of Dengue Virus Type 1 to 4 Strains Used for the Development of CBER/FDA RNA Reference Reagents and WHO International Standard Candidates for Nucleic Acid Testing

**DOI:** 10.1128/genomeA.01583-15

**Published:** 2016-02-11

**Authors:** Germán Añez, Daniel A. Heisey, Evgeniya Volkova, Maria Rios

**Affiliations:** Laboratory of Emerging Pathogens, Division of Emerging and Transfusion Transmitted Diseases, Office of Blood Research and Review, Center for Biologics Evaluation and Research, U.S. Food and Drug Administration, Silver Spring, Maryland, USA

## Abstract

Dengue virus (DENV), a member of the *Flaviviridae* family, is the most common and clinically significant arbovirus in the world and is endemic in more than 100 countries. Here, we report the complete sequences of four DENV serotypes used in the development of the CBER/FDA RNA reference reagents and WHO International Standard candidates for nucleic acid testing.

## GENOME ANNOUNCEMENT

Dengue virus is the leading cause of arboviral disease in the world, with more than 390 million cases/year in tropical and subtropical regions of the Americas, Asia, Africa, and Pacific islands (1). There are four dengue serotypes (DENV-1 to DENV-4), which are transmitted by *Aedes* mosquitoes, mainly *A. aegypti*. While up to 75% of DENV infections are asymptomatic, some patients develop an influenza-like illness that may progress to a life-threatening condition known as severe dengue (dengue hemorrhagic fever/dengue shock syndrome). To date, there are no vaccines or specific antiviral treatments for DENV (2). These viruses have been detected in asymptomatic blood donors and transmitted by blood transfusion and organ transplantation, threatening the safety of the blood and organ supply (3–6). Laboratory diagnosis of DENV infection is made by viral isolation, serology, detection of viral antigens (i.e., NS1 antigen), or by nucleic acid testing (NAT), which is considered the most sensitive detection method (7). Although there are FDA-approved serological and NAT assays for diagnosis of infection with DENV, no blood screening assays are currently available (8, 9). With the aim of assisting assay developers and to streamline the process of regulatory evaluation for assays seeking licensure, we have prepared CBER/FDA reference reagents and the first WHO International Standard candidates using four DENV laboratory strains.

We describe here the complete genomes of DENV-1 strain Hawaii (collected in Hawaii in 1944), DENV-2 strain New Guinea C (collected in Papua New Guinea in 1944), and DENV-3 strain H87 and DENV-4 strain H241 (both collected in the Philippines in 1956). After isolation from human specimens, these strains underwent extensive laboratory manipulations, becoming adapted to cell culture and animals. Cell culture supernatants from the fourth passage of the viruses in C6/36 cells were used for RNA extraction using the QIAamp Viral RNA minikit (Qiagen). cDNA was produced with SuperScript III reverse transcriptase (Invitrogen) and DENV type-specific primers, and overlapping PCR products were produced with TaKaRa LA Taq DNA polymerase (TaKaRa). Alternatively, these two steps were combined using the Titan One Tube RT-PCR system (Roche). Amplicons were sequenced by the Sanger method. 5′- and 3′-untranslated regions (UTR) were resolved by cyclization of virus RNA followed by reverse transcriptase PCR. The sequences were assembled and analyzed using Sequencher version 5.2.4 (GeneCodes Corp.).

The total lengths of the genomes were as follows: DENV-1, 10,736 nucleotides (nt); DENV-2, 10,723 nt; DENV-3, 10,696 nt; and DENV-4, 10,664 nt. 5′- and 3′-UTR sequences were 95/462 nt, 96/451 nt, 94/429 nt, and 101/400 for DENV-1 to DENV-4, respectively.

We found a number of differences between our sequences and those published for reference strains (GenBank accession numbers for DENV-1 to DENV-4: EU848545, AF038403, M93130, AY947539), including nt deletions and insertions in the 3′-UTR and double peaks in the sequencing electropherograms, suggesting the presence of more than one viral population containing two different nt at a certain position.

### Nucleotide sequence accession numbers.

The complete genomes of the DENV-1, DENV-2, DENV-3, and DENV-4 strains have been submitted to GenBank under the accession numbers KM204119, KM204118, KU050695, and KR011349, respectively.
